# Assessing the fish fauna diversity and abundance at Aegean and Ionian seas, with emphasis on certain NIS fish species via Scientific diving and Visual Census

**DOI:** 10.1371/journal.pone.0294770

**Published:** 2023-11-29

**Authors:** Thodoros E. Kampouris, Sofia Pappou, Zinovia Erga, Vasilis Kouloumperis, Ioannis E. Batjakas

**Affiliations:** 1 Department of Marine Sciences, University of the Aegean, University Hill, Lesvos Island, Mytilene, Greece; 2 Astrolabe-Marine Research, Lesvos Island, Mytilene, Greece; 3 Oceanides-Institute of Marine Research & Education; 4 16, Athanasiou Diakou str., 17343, Athens, Greece; Ocean Frontier Institute, CANADA

## Abstract

The Mediterranean Sea and its exceptional biodiversity suffer from non-indigenous (NIS) and invasive species. These result from Lessepsian migration and human activities. Species with the highest negative impact include *Pterois miles*, *Lagocephalus sceleratu*s and *Fistularia commersonii*. The current study assessed the distribution and abundance of these three species in the Aegean and Ionian Seas in the context of the regional diversity of ichthyofauna. Using Scientific Diving and Visual Census, we focused on NIS fish fauna, and surveyed most of the areas where the occurrence or establishment of NIS had been documented. Emphasis was given to regions with limited available data. This study assessed the homogeneity of diversity and abundance of the fish species at the diving sites; assessed the most dominant species per region; and calculated relevant biodiversity indexes. Our results suggest that the south and east parts of the Aegean Sea are the most affected by the establishment of NIS. Samos Island is concluded to be an important region for the expansion of NIS to the northern parts of the Aegean Sea. Also, both the Saronikos Gulf and the whole area of the Peloponnese seem to have a pivotal role in the expansion of distribution of NIS from the east Mediterranean sub-basin to the west sub-basin. The genus *Siganus* seems to have very high abundances and population densities in certain areas, and therefore its establishment could have negative impacts in these regions. We conclude that scientific diving is not appropriate for monitoring the population status of *L*. *sceleratus* and we propose more appropriate methodologies such as the use of baited cameras and ROV’s to be used in the future.

## Introduction

In the Mediterranean region, human activities such as marine transportation, aquaculture, aquarium trade and the seafood industry assist the introduction of marine species [1–5]. A recent review showed that during a three-year period (2017–2019), twenty new exotic fishes were reported form the Mediterranean Sea, and for the time being 666 exotic marine species (fauna and flora) should be considered as established [6]. However, besides the well-acknowledged negative effects of biological invasions, a recent study highlighted the fact that some non-indigenous species may have positive impacts [7]. Among the non-indigenous marine fishes with extend distributions and with documented negative impacts in the Mediterranean Sea are: (i) *Pterois miles* (Bennett, 1828), (iii) *Lagocephalus sceleratus* (Gmelin, 1789) and (iii) *Fistularia commersonii* Rüppell, 1838.

*Pterois miles* is native to Indo-Pacific region. The first Mediterranean report was in 1991 from Israeli coasts [8]. However, the actual invasion began in 2012–2013 when the species was recorded off Lebanon and Cyprus, see Dimitriou et al. (2019) [9] and references within for further details. Since then, the lionfish, is rapidly expanding its distribution and establishment in Mediterranean Sea, although most records occur in the eastern part of the basin, with sporadic occurrence or lack of records in the central and western part [10, 11]. The study of Dimitriadis et al. (2020) [12] provides the most updated information, up to 2020, on the species distribution expansion. Studies on the lionfishes’ population genetics revealed that the introduction in the Mediterranean Sea happened through successive occurrences and not by a single event [9, 13]. It seems that the invasive lionfishes in the Mediterranean are chiefly of Red Sea origin [9, 13], but some individuals form Cyprus originated from the Indian Ocean, suggesting a different (to the Suez Canal) pathway of introduction [9]. In Mediterranean waters, lionfishes showed an extend period of reproduction, that seems to peak during the summer. Moreover, each female is able to complete its reproduction cycle multiple times during each season [14]. Several studies documented that its feeding behaviour and diet habits can set significant alterations on regional food webs in the Mediterranean basin [15–17]. In Rhodes Island, Greece the predominantly prey was fish species (94.7% in biomass and 78.5% in number) [15]. Similar results were presented by Savva et al. (2020) off Cyprus [16]. On the contrary, the study of D’ Agostino et al. (2020) also from Cypriot waters, stated that *P*.*miles* preferred small sized benthic invertebrates [17].

*Lagocephalus sceleratus-* Silver-cheeked toadfish, is one of the nine allochthonous and one native species of Family Tetraodontidae, that were recorded in European waters thus far [18, 19]. It is a Lessepsian migrant, occurring naturally at the Red Sea and Indo-Pacific, inhabiting shallow coral reefs [20]. It invaded many sub-basins and seas of Mediterranean waters [18, 19]-including the Sea of Marmara [21], and in many areas, it is considered as established [19]. The first record from Greece was in 2005 from Rhodes Island, south-east Aegean Sea [22]. In Egypt the species’ life span was estimated around 7 years [23], while other studies from Turkish coast extends it at 10 years [24]. In Levantine Sea the spawning takes place from mid-spring to mid-summer depending on region [25–27]. The Silver-cheeked toadfish prefers sandy bottoms [27–29] but seagrass meadows are preferable habitats for reproductive adults, at least at Aegean Sea [27]. It feeds on crustaceans [23] and it seems to have an ontogenetic shift since larger individuals prefer cephalopods such as the common octopus and the common cuttlefish [28]. A recent study showed that *L*. *sceleratus* feeds on other non-indigenous fauna like the lionfish (*P*. *miles*), rabbitfishes (*Siganus* spp.), the Red Sea goatfish [*Parupeneus forsskali* (Fourmanoir & Guézé, 1976)], and the long-spine sea urchin (*Diadema setosum* Leske, 1778) [30].

*Fistularia commersonii*—bluespotted cornetfish, is one of the two Fistularia species -other being *F*. *petimba* reported from Mediterranean waters [31–33], already recorded from the Sea of Marmara [34]. The bluespotted cornetfish is broadly distributed at the tropical-subtropical Indo-Pacific waters and at the Red Sea [19, 35] and introduced at east Mediterranean Basin through the Suez Canal [36]. Besides Mediterranean Sea, *F*. *commersonii* is introduced in Californian waters [37]. It is one of the most successful colonisers, since it is a species with strong dispersal potentiality [38] and in European waters, it is continuously reported from the whole Mediterranean Sea [39]. It is acknowledged that the invasion of *F*. *commersonii* resulted from a single incident [40]. Furthermore, *F*. *commersonii* inhabits many different habitats, has a multiple reproduction pattern [41], grows fast [42] and may live as 5 years [43]. Also, as a piscivorous species, in Mediterranean, preys upon fish species such *Spicara smaris* (Linnaeus, 1758) and *Boops boops* (Linnaeus, 1758) [41, 44–46] or upon shoaling fishes at the water column [45]. Larger fish, lengthwise, tend to feed on larger prey [41, 45], presenting similarities with Japanese specimens [47]. In Greek waters the bluespotted cornetfish was firstly reported from Rhodes Island [48] and it is established in south Aegean Sea [44, 49], and it is a frequent component of the coastal fisheries [50]. Also, is considered established in the Mediterranean basin, e.g., France, Lebanon, among many others [51, 52]. For the most updated information on the species’ occurrences in Greek territorial waters see ELNAIS (2023) [53].

The present study aims to assess the regional distribution and abundance of native and NIS ichthyofauna in Aegean and Ionian seas, via Scientific Diving and Visual Census. The fieldwork took place in several locations of the Hellenic seas covering regions where NIS are considered as established and at regions where limited scientific knowledge exists (i.e., Peloponnese). The fixed-time diving protocol was used, and each dive was 45 minutes. Empasis was given at three invasive fish species: the lionfish, the silver-cheeked toadfish, and the bluespotted cornetfish. Furthermore, the current study assessed the total fish fauna diversity, calculated relevant biodiversity indexes and assessed the ecological similarities between sampling stations and sites.

## Materials and methods

### Scientific diving methodology and quality control

Scientific diving surveys conducted in both Aegean and Ionian seas in 2021 and 2022. The sampling sites and stations were selected based on three factors: (i) to cover most of the areas where non-indigenous occur and are established (ii) the selected sites were set in a north-south axis having in mind the influence of the warm and saline water currents of Levantine Sea origin, influencing the east Aegean Sea [54], and (iii) to cover areas with limited or no data available such as Peloponnese, see Katsanevakis (2021) [55]. Regions such as the Thracian Sea (NE Aegean Sea), the NW Aegean Sea were excluded, since there are very limited occurrences of non-indigenous fish fauna. Moreover, the region of Crete was excluded as well. It is considered to be well studied.

During the current study, 21 diving surveys were performed. At the North Aegean Sea (NAS, thereafter), diving was conducted at Lesvos Islands in two sites and at Samos Island, two sites as well. The South Aegean Sea (SAS, thereafter) the diving sites included: (i) The Saronikos Gulf, SW Aegean Sea, one dive. The regions of Attica and Saronikos Gulf are acknowledged as an important area for the expansion of non-indigenous species to Greek west coasts and essentially to their dispersal to Ionian and the rest Mediterranean basins [56], (ii) The region of Cyclades and Santorini Island, two dives, (iii) the region of Dodecanese and the islands of Kalymnos and Nisyros, with three and two stations respectively. Almost all gulfs of the region of Peloponnese were surveyed at the framework of this this study. The NW Ionian coasts, of Peloponnese, were excluded, due to the practical absence of available data concerning the status of non-indigenous fish, excluding the study of Katsanevakis (2021) [55]. The SE diving sites of Peloponnese were grouped as SAS for further analysis, while the reaming SW and W sites were grouped as Ionian Sea (IS, thereafter). The last region was the island of Kefalonia with two diving sites. Table 1 lists all the available information regarding the region, the area of each survey, the coordinates and code (e.g., LES1) that was given for all diving sites. In each site, two different habitats were surveyed: (i) seagrass beds, mainly targeting the preferred habitat of *F*. *comersonii* and (ii) rocky bottoms or biogenic reefs, targeting the preferred habitat of *P*. *miles* and *L*. *sceleratus*.

**Table 1:** The list of diving stations. Information is provided about the site, the area, the eco-region, the code, and the coordinates.

Sampling station	Sampling site	Eco-region	Code	Coordinates
Ag. Vasilios	Lesvos Island	NE Aegean	LES1	38°58’15.2"N
				26°32’31.1"E
Akra Valvi	Lesvos Island	NE Aegean	LES2	38°59’26.3"N
				26°32’39.6"E
Kokkari 1	Samos Island	NE Aegean	SAM1	37°46’52.4"N
				26°53’33.1"E
Kokkari 2	Samos Island	NE Aegean	SAM2	37°46’52.7"N
				26°53’36.9"E
Pnigmenos reef	Kalymnos Island	SE Aegean	KAL1	36°59’30.2"N
				26°54’49.6"E
Pnigmenos Bay	Kalymnos Island	SE Aegean	KAL2	36°59’34.6"N
				26°54’58.4"E
Therma	Kalymnos Island	SE Aegean	KAL3	36°56’18.7"N
				26°59’15.5"E
Pachia ammos	Nisyros Island	SE Aegean	NIS1	36°35’19.7"N
				27°12’40.5"E
Loutra	Nisyros Island	SE Aegean	NIS2	36°36’49.1"N
				27°09’15.3"E
Lion’s face	Santorini Island	S Aegean	SAN1	36°21’41.7"N
				25°24’08.9"E
Balos reef	Santorini Island	S Aegean	SAN2	36°21’41.1"N
				25°24’19.1"E
Proto Limanaki	Saronikos Gulf	SW Aegean	SAR1	37°47’59.8"N
				23°47’19.9"E
Kiveri Bay	Peloponnese	SW Aegean	PEL1	37°31’33.7"N
				22°43’53.0"E
Tolo	Peloponnese	SW Aegean	PEL2	37°30’15.8"N
				22°51’52.2"E
Korakas	Peloponnese	SW Aegean	PEL3	36°26’28.3"N
				23°05’32.4"E
Stoupa	Peloponnese	Ionian	PEL4	36°51’03.0"N
				22°15’27.8"E
Kardamili	Peloponnese	Ionian	PEL5	36°52’53.5"N
				22°13’59.8"E
Agios Andeas 1	Peloponnese	Ionian	PEL6	37°40’00.1"N
				21°18’13.9"E
Agios Andeas 2	Peloponnese	Ionian	PEL7	37°40’03.0"N
				21°18’08.2"E
Skala 1	Kefalonia	Ionian	KEF1	38°05’51.3"N
				20°48’30.3"E
Skala 2	Kefalonia	Ionian	KEF2	38°05’27.8"N
				20°48’14.6"E

Underwater Visual Census is widely applied when assessing the composition and abundance of fish communities; it can be done via SCUBA or via free diving and in combination with the use of obtaining digital material (video and photos) [57]. There are several techniques that can be used, including the fixed-time survey (FTS) that we performed here; the specific technique is low cost, and it is proposed when studying species composition and spatial distribution like the current study [58]. The bottom time in all dives was to 45 minutes, the depth varied depending on the site’s topography, bathymetry, and access point (shore or boat dive). In each dive three people were involved, one was the safety-back up diver and the remaining two people had specific tasks, one was responsible for obtaining digital material and the other was keeping record of the fish species composition along with an estimate of their respective abundances.

The fish taxa were identified based on their external morphology *in situ*. However, two validation procedures were done by two scientists independently in order to secure proper identification of taxa, by checking the complete photographic/videographic data for each station. In the case of disagreement, the taxa validation was performed by project’s PI. If still some degree of uncertainty remained, then the taxa were recorded to the upper taxonomic level (e.g., genus or family).

### Biodiversity indexes

Within the framework of the present study, four key biodiversity indexes regarding the ichthyofauna (in total) were calculated based on the relative abundance of each species. These were: (i) Margalef, (ii) Shannon-Wiener, (iii) Pielou and (iv) Simpson.

**(i) Margalef (d) index.**

d=(S‐1)/logN

*S*, the species number and *N*, the number of individuals. This index expresses the number of species in the sample while it is also affected by the size of the sample.

**(ii) Shannon-Wiener index.**

H′=‐Σipi(logpi)

*pi* the relative abundance (could be biomass as well) of a species (i) in each sample. This index is used for the analysis of biocommunities and expresses both the number of species and the degree of distribution uniformity individuals in the various species within a system. When all individuals belong to one species, then the index takes the minimum possible value, (i.e., zero). When all species have the same number of individuals, theoretically the index is infinite.

**(iii) Pielou index.**

J′=H′/log(S)

*Η΄* is the value of the Shannon-Wiener diversity index. This index expresses how individuals are distributed across species. The maximum value of the index is 1, when the distribution is uniform.

**(iv) Simpson index.**

1‐λ=1‐Σ[ni(ni‐1)/Ν(Ν‐1)]

This index describes the heterogeneity of the samples. The advantage of this index is that it does not depend on the variation of species abundance. However, when the number of samples is small (less than 10), its values are often underestimated.

### Statistical analysis

An initial comparison was conducted using paired Student’s t-tests, for repeated abundances measurements of fish fauna with sampling stations as replicates; excluding the samples from Saronikos Gulf since only one dataset was available. Since no statistical differences were observed, the data were pooled at site level (Lesvos, Samos, etc.). A multidimensional scaling (MDS) analysis, of square-root transformed data, using Primer 6 software package [59] was conducted to visualise similarities in fish assemblages between sampling stations across study sites, and across the ecoregions (NAS: North Aegean Sea, SAS: South Aegean Sea, and IS: Ionian Sea). Similarity Percentages analyses were conducted in Primer 6, using the SIMPER methodology to identify the sites with the greatest similarity. The same procedures were performed only for the non-exotic species.

No permits and permissions were required for conducting the present study. All diving sites were located in areas where recreational diving is permitted. No further installations and other instruments (i.e., transects, threads etc.) were set. All diving spots were not associated with any Marine Protected Area(s) or Sites of Archaeological Interest.

## Results

Based on the current findings it can be said that neither SCUBA diving nor snorkelling along with visual census are suitable sampling methodologies, concerning the assessment of the spatial distribution and abundance of *L*. *sceleratus* since no individuals were reported at all sites and stations, despite the fact that a plethora of previous studies refer the species occurrence or establishment at the same regions. The recent study of Katsanevakis (2021) [55], confirms the above statement.

Totally, 67 fish taxa belonging to 21 families were recorded in all stations and sites. The family with the highest occurrences was Sparidae. The species with the most occurrences, being present to all sites and stations was *Serranus scriba* (Linnaeus, 1758), followed by *Diplodus sargus* (Linnaeus, 1758) and *D*. *vulgaris* (Geoffroy Saint-Hilaire, 1817) being absent only from one site. The most frequent and abundant non-indigenous species were the rabbitfishes *Siganus luridus* (Rüppell, 1829) and *S*. *rivulatus* Forsskål & Niebuhr, 1775, being absent only from Lesvos Island (Table 2).

**Table 2:** The composition of the fish fauna in all sampling sites and stations in terms of presence/absence.

Family	Species	Sampling Sites and Stations																				
		Lesvos		Saronikos	Peloponnese							Kefalonia		Nisyros		Santorini		Samos		Kalymnos		
		1	2	1	1	2	3	4	5	6	7	1	2	1	2	1	2	1	2	1	2	3
Atherinidae	*Atherina sp*.	a	a	a	p	a	a	a	a	a	a	a	a	a	a	a	a	a	a	a	a	a
Apogonidae	*Apogon imberbis*	a	a	a	a	a	a	a	a	a	a	a	a	a	p	p	p	p	p	p	p	p
Blenniidae	*Parablennius sp*.	a	a	a	a	a	a	a	a	a	a	a	a	p	a	a	p	a	a	a	a	a
Bothidae	*Bothus podas*	a	a	a	a	a	a	a	a	a	a	a	a	a	p	a	a	a	a	a	a	a
Callanthiidae	*Callanthias ruber *	p	a	a	a	a	a	a	a	a	a	a	a	a	a	a	a	a	a	a	a	a
Carangidae	*Caranx crysos*	a	a	a	a	a	a	a	a	a	a	a	a	a	p	a	a	a	a	a	a	a
	*Lichia amia*	a	a	a	a	a	a	a	a	a	a	p	a	a	a	a	a	a	a	a	a	a
Engraulidae	*Engraulis encrasicolus*	a	a	a	a	a	a	a	a	a	a	a	a	a	a	a	a	p	a	a	p	a
Fistulariidae	*Fistularia commersonii*	a	a	a	a	a	a	p	p	a	a	a	a	a	p	a	a	a	p	p	a	a
Gobiidae	*Gobius bucchichi*	a	a	a	a	a	a	a	a	a	a	a	a	a	a	a	a	a	p	a	a	a
	*Gobius sp*.	a	a	a	a	a	a	a	a	a	a	a	a	a	a	a	a	a	a	a	p	p
Holocentridae	*Sargocentron rubrum*	a	a	a	a	a	a	a	a	a	a	a	a	p	p	a	a	a	a	a	a	p
Labridae	*Centrolabrus melanocercus*	a	a	a	a	a	a	a	a	a	a	a	a	a	a	p	a	p	p	p	a	a
	*Coris julis*	p	p	p	p	p	p	p	p	p	a	a	a	p	p	p	p	p	p	p	p	p
	*Pteragogus trispilus*	a	a	p	a	a	a	a	a	p	a	a	a	a	a	a	a	a	a	a	a	a
	*Symphodus cinereus*	a	a	a	a	a	a	a	a	a	a	a	a	a	a	a	p	a	a	a	a	a
	*Symphodus mediterraneus*	a	a	a	a	a	p	a	a	a	a	a	a	p	a	a	a	p	p	p	p	a
	*Symphodus roissali*	a	a	a	a	a	p	a	a	p	a	a	a	a	a	a	a	a	a	a	a	a
	*Symphodus rostratus*	a	a	a	a	a	a	a	a	a	a	a	a	a	a	a	p	A	a	a	a	a
	*Symphodus tinca*	p	p	p	p	p	p	p	p	p	p	p	a	a	a	p	a	p	p	p	p	a
	*Thalassoma pavo*	p	p	a	a	p	p	p	a	p	p	p	a	p	p	p	p	p	p	p	p	p
	*Xyrichtys novacula*	a	a	a	a	p	a	a	a	a	a	a	a	a	a	a	a	a	a	a	a	a
Monacanthidae	*Stephanolepis diaspros*	a	a	a	a	a	a	a	a	a	a	a	a	a	a	p	p	a	a	a	a	a
Mugilidae	*Mugil cephalus*	a	a	a	a	a	a	a	a	a	a	a	a	a	a	a	a	a	a	a	p	a
Mullidae	*Mullus surmuletus*	a	p	p	p	p	p	p	p	p	p	p	p	p	p	p	p	p	p	p	p	p
	*Parupeneus forsskali*	a	a	a	a	a	a	a	a	a	a	a	a	p	p	a	p	a	a	p	p	p
	*Upeneus sp*.	a	a	p	a	a	a	a	a	a	a	a	a	a	a	a	a	a	a	a	a	a
Muraenidae	*Muraena helena*	a	p	a	a	a	a	a	a	a	a	a	a	a	a	a	a	a	a	a	a	a
Pomacentridae	*Chromis chromis*	p	p	p	p	p	p	p	p	p	p	p	a	p	p	p	p	p	p	p	p	p
Scaridae	*Sparisoma cretense*	a	p	a	p	p	p	p	a	p	p	p	p	p	p	p	p	p	p	p	p	p
Sciaenidae	*Sciaena umbra*	a	a	a	a	p	a	a	a	a	a	a	a	a	a	a	a	a	a	a	a	a
Scombridae	*Thunnus sp*.	p	a	a	a	a	a	a	a	a	a	a	a	a	a	a	a	a	a	a	a	a
Scorpaenidae	*Pterois miles*	a	a	a	a	p	a	p	p	a	p	p	p	p	p	p	p	p	a	p	p	p
	*Scorpaena maderensis*	a	a	a	a	a	a	a	a	a	a	a	a	a	a	a	p	p	p	p	p	p
	*Scorpaena porcus*	a	a	a	a	a	a	a	a	a	a	a	a	a	a	a	a	p	a	a	a	a
	*Scorpaena scrofa*	p	a	a	a	a	a	a	a	a	a	a	a	a	a	a	a	a	a	a	a	a
	*Scorpaena sp*.	a	a	a	a	a	a	p	a	a	a	a	a	p	a	a	p	a	a	a	a	a
Siganidae	*Siganus luridus*	a	a	p	p	p	p	p	p	p	p	p	p	p	p	p	p	p	p	p	p	p
	*Siganus rivulatus*	a	a	p	p	p	p	p	p	p	p	p	p	p	p	p	p	p	p	p	p	p
Serranidae	*Anthias anthias*	p	a	a	a	a	a	a	a	a	a	a	a	a	a	a	a	a	a	p	p	a
	*Epinephelus aeneus*	a	a	a	p	a	a	a	a	a	a	a	a	a	a	a	a	a	a	a	a	a
	*Epinephelus costae*	a	a	p	a	p	a	p	p	p	a	a	a	p	p	p	p	p	p	a	a	a
	*Epinephelus marginatus *	p	a	a	a	p	p	p	p	p	a	p	a	p	p	p	p	p	p	a	a	a
	*Serranus cabrilla*	p	p	p	p	p	p	p	p	p	a	a	p	p	p	p	p	p	a	p	p	p
	*Serranus scriba*	p	p	p	p	p	p	p	p	p	p	p	p	p	p	p	p	p	p	p	p	p
Sparidae	*Boops boops*	a	a	a	a	a	a	a	a	a	a	a	a	a	p	p	p	p	p	a	a	p
	*Dentex dentex*	a	a	a	a	p	a	a	a	p	a	a	a	a	a	p	p	a	a	a	a	a
	*Diplodus annularis*	a	a	a	p	a	a	a	a	a	a	a	a	p	p	p	a	a	a	a	a	a
	*Diplodus puntazzo*	a	a	a	a	a	a	a	a	a	a	a	a	a	a	p	p	a	a	p	a	a
	*Diplodus sargus*	p	p	p	p	p	p	p	a	p	p	p	p	p	p	p	p	p	p	p	p	p
	*Diplodus vulgaris*	p	p	p	p	p	p	p	a	p	p	p	p	p	p	p	p	p	p	p	p	p
	*Lithognathus mormyrus*	a	a	p	a	a	a	a	a	a	a	a	a	p	a	a	a	a	a	a	a	a
	*Oblada melanura*	p	a	a	a	p	a	a	p	p	a	a	a	p	p	p	p	p	a	p	a	p
	*Pagellus erythrinus*	a	a	p	a	a	a	a	a	a	a	a	a	a	a	a	a	a	a	a	a	a
	*Sarpa salpa*	p	p	p	a	p	a	a	a	a	a	a	a	a	a	p	p	p	p	p	p	p
	*Sparus aurata*	p	a	p	a	a	a	a	a	a	a	a	a	a	a	a	a	a	a	a	a	a
	*Spicara flexuosum*	a	a	a	a	a	a	a	a	a	a	a	a	p	a	p	p	a	p	p	a	a
	*Spicara maena*	a	a	a	a	a	a	a	a	a	a	a	a	p	a	p	p	a	p	p	a	a
	*Spicara smaris*	a	a	p	a	p	a	a	a	a	a	a	a	p	p	a	a	a	a	a	a	a
	*Spicara sp*.	a	a	a	a	a	a	a	a	a	a	a	a	p	p	p	p	a	a	a	a	a
	*Spondyliosoma cantharus*	a	a	p	p	a	p	a	a	a	a	a	a	a	a	a	a	a	a	a	a	a
Sphyraenidae	*Sphyraena sp*.	a	a	a	a	a	a	a	a	a	a	a	a	a	a	p	p	a	a	a	a	a
Synodontidae	*Synodus saurus*	a	a	p	a	p	a	a	a	a	a	a	a	a	a	a	p	a	a	a	a	a
Trachinidae	*Trachinus draco*	a	a	a	a	p	a	a	a	a	a	a	a	a	a	a	a	a	a	a	a	a

a: Absence, p: Presence.

The values of the assessed biodiversity indices are listed at Table 3. The relative information is presented for each sampling station.

**Table 3:** Τhe values of biodiversity indexes in all sampling stations of the present study.

	S	N	d	J’	H’(loge)	1-Lambda’
**LES1**	16	84.03	3.39	0.84	2.34	0.88
**LES2**	12	61.29	2.67	0.83	2.06	0.83
**SAR1**	20	71.47	4.45	0.86	2.57	0.88
**PEL1**	15	94.85	3.08	0.72	1.94	0.77
**PEL2**	23	98.72	4.79	0.88	2.76	0.92
**PEL3**	16	57.62	3.70	0.81	2.24	0.83
**PEL4**	17	88.20	3.57	0.87	2.45	0.89
**PEL5**	13	58.36	2.95	0.89	2.27	0.88
**PEL6**	18	100.74	3.69	0.86	2.50	0.89
**PEL7**	11	47.14	2.60	0.91	2.18	0.89
**KEF1**	13	48.04	3.10	0.91	2.33	0.90
**KEF2**	9	28.28	2.39	0.96	2.12	0.90
**NIS1**	26	220.20	4.63	0.86	2.79	0.92
**NIS2**	27	144.52	5.23	0.90	2.96	0.94
**SAN1**	27	146.37	5.21	0.89	2.94	0.93
**SAN2**	32	190.97	5.90	0.85	2.95	0.94
**SAM1**	24	122.12	4.79	0.92	2.94	0.95
**SAM2**	23	92.68	4.86	0.91	2.87	0.94
**KAL1**	24	132.48	4.71	0.92	2.92	0.94
**KAL2**	22	111.41	4.46	0.92	2.83	0.94
**KAL3**	20	78.16	4.36	0.93	2.79	0.94

**Fig 1:**
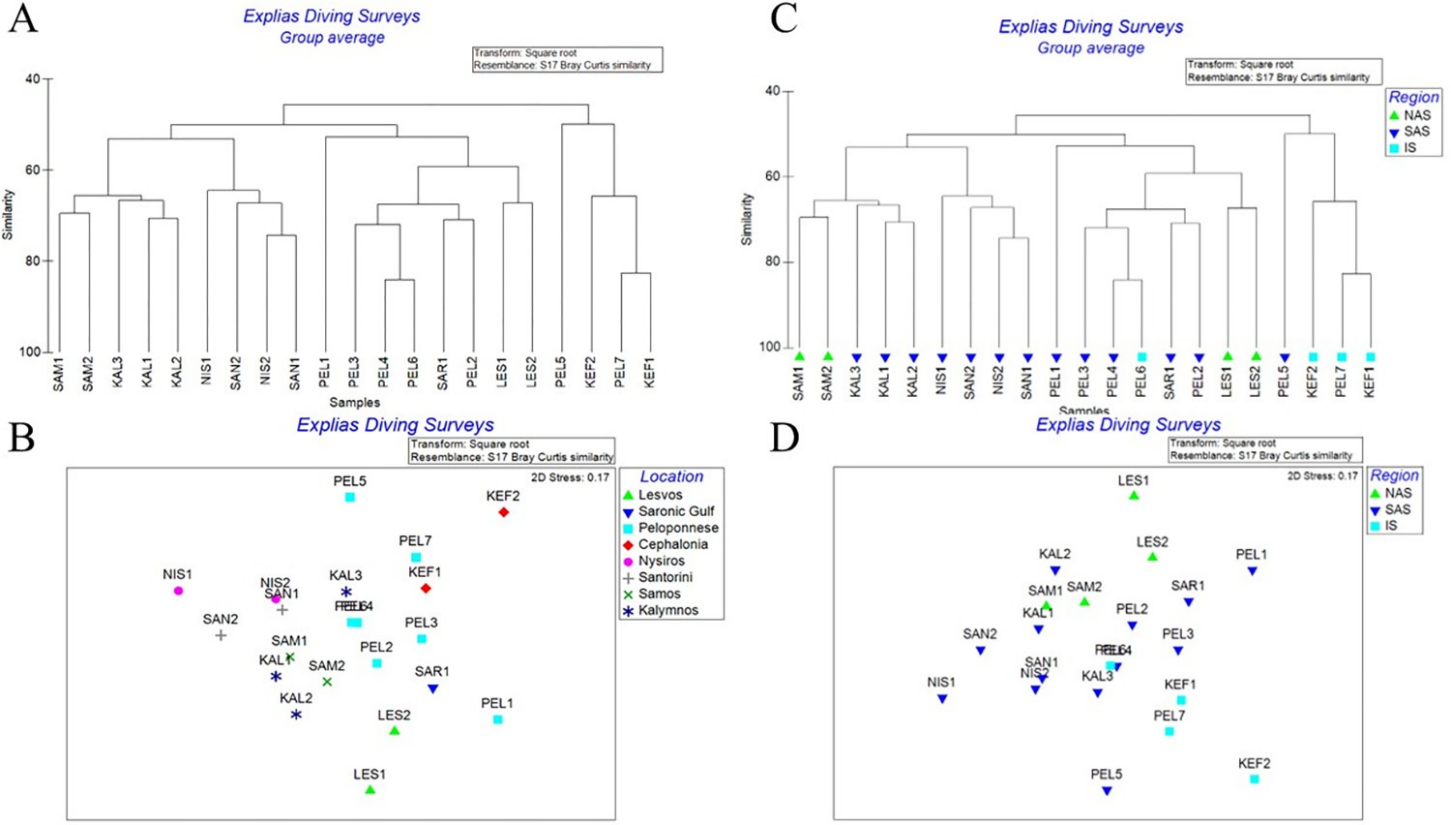
Cluster and MDS analysis. (Α) Cluster analysis (% similarity) of sites and sampling stations (Β) MDS Analysis of sites and sampling stations. (C) Cluster analysis (% similarity) of sites and sampling stations, per ecoregion. (D) MDS Analysis of sites and sampling stations, per ecoregion. (NAS: North Aegean Sea, SAS: South Aegean Sea, IS: Ioanian Sea).

Regarding the ichthyofauna in total, the similarity among stations ranged from >40% to >75%. The most similar stations were PEL4-PEL6 (Stoupa—Ag. Andreas1, Katakolo) and PEL7-KEF1 (Ag. Andreas2 –Skala1) with similarity >75% (Fig 1A). Furthermore, the stations of Lesvos Island are forming a separate, yet not distant, group from the remaining sites and stations (Fig 1B). On the contrary, Samos Island though that geographically is considered as an area of the north-eastern Aegean Sea, its composition of ichthyofauna is fitting well with the species’ composition of regions and aeras of south Aegean Sea like Kalymnos Island, and to a lesser extend with Nisyros and Santorini islands (Fig 1C). Noteworthy, is the fact that the diving sites of Lesvos Island along with Saronikos Gulf and some stations of Peloponnese are forming several subgroups and a larger group of >50% similarity (Fig 1C and 1D).

The diving stations among sites were very similar to each other. The similarity percentages were from >60% to >71%. The site with the most similar survey sites was Santorini with 71.12% and that with the least similarity was Peloponnese with 60.03%. The values of similarities for the remaining sites; presented in decreasing order, were Samos (69.53%), Nisyros (68.06%), Kalymnos (68.01%), Lesvos (67.3%) and Kefalonia (66.52%). Table 4 presents the Average Similarity of the most significant species per region and Table 5 the Average Abundance per region always in respect to the most significant species.

**Table 4:** The Average Similarity among sampling sites, based on the findings of the present study. Marked with bold font are the most dominant species.

Species	Average Similarity						
	PEL	KEF	LES	KAL	NIS	SAM	SAN
*Anthias anthias*	-	-	-	2.99	-	-	-
*Apogon imberbis*	-	-	-	2.75	-	3.61	1.97
*Boops boops*	-	-	-	-	1.98	2.28	10.9
*Centrolabrus melanocercus*	-	-	-	-	-	1.86	-
*Chromis chromis*	**18.99**	-	**30.77**	**11.85**	**14.51**	**12.83**	**15.69**
*Coris julis*	3.31	-	6.6	3.95	3	5.1	3.41
*Diplodus sargus*	2.67	8.29	5.33	4.27	-	4.27	4.96
*Diplodus vulgaris*	3.18	9.45	5.33	3.84	2.57	2.28	3.14
*Epinephelus costae*	-	-	-	-	1.65	-	-
*Mullus surmuletus*	1.93	4.54	-	-	1.82	3.36	1.45
*Oblada melanura*	-	-	-	-	4.42	-	2.52
*Parupeneus forsskali*	-	-	-	2.88	2.33	-	-
*Pterois miles*	-	-	-	2.15	1.65	-	-
*Sarpa salpa*	-	-	-	3.86	-	8.06	-
*Serranus cabrilla*	1.96	-	-	1.43	-	-	1.45
*Serranus scriba*	3.32	7.41	-	-	1.65	2.46	1.45
*Siganus rivulatus*	7.1	**12.57**	-	7.23	5.48	4.56	7.26
*Siganus luridus*	7.79	**12.57**	-	4.37	5.48	3.09	7.26
*Sparisoma cretense*	2.67	9.08	-	5.92	3.68	5.89	2.96
*Spicara maena*	-	-	-	-	-	-	3.75
*Spicara smaris*	-	-	-	-	4.59	-	-
*Spicara sp*.	-	-	-	-	3.24	-	2.52
*Symphodus tinca*	2.78	-	7.91	-	-	-	-
*Thalassoma pavo*	-	-	4.96	4.32	4.75	3.61	2.96

The Overall Similarity per diving site in increasing order: PEL-60.03%, KEF-66.52%, LES-67.3%, KAL-68.01% NIS-68.06%, SAM-69.53% and SAN-71.12%

**Table 5:** The Average Abundance of the most dominant species among sampling sites, based on the findings of the present study. Marked with yellow are the most dominant species.

Species	Average Abundance						
	PEL	KEF	LES	KAL	NIS	SAM	SAN
*Anthias anthias*	-	-	-	8.37	-	-	-
*Apogon imberbis*	-	-	-	4.83	-	7.11	3.89
*Boops boops*	-	-	-	-	17.61	4.76	10.9
*Centrolabrus melanocercus*	-	-	-	-	-	2	-
*Chromis chromis*	**19.03**	-	**22.36**	**13.83**	**32.59**	**14.31**	**26.46**
*Coris julis*	3.71	-	5.27	4.99	9.9	6.77	6.41
*Diplodus sargus*	3.72	3.38	3.94	5.41	-	5.03	8.95
*Diplodus vulgaris*	4.09	3.61	3.94	5.89	6.82	3.46	6.98
*Epinephelus costae*	-	-	-	-	3.3	-	-
*Mullus surmuletus*	1.93	2.09	-	-	3.46	3.8	3.29
*Oblada melanura*	-	-	-	-	9.03	-	10.78
*Parupeneus forsskali*	-	-	-	3.21	4.99	-	-
*Pterois miles*	-	-	-	2.55	3.85	-	-
*Sarpa salpa*	-	-	-	4.64	-	8.8	-
*Serranus cabrilla*	2.16	-	-	1.79	-	-	3.29
*Serranus scriba*	3.23	2.83	-	-	3.08	3.44	2.88
*Siganus rivulatus*	7.92	**4.9**	-	8.7	11.12	5.19	12.25
*Siganus luridus*	8.24	**4.9**	-	5.54	11.12	3.72	12.25
*Sparisoma cretense*	2.59	3.53	-	7.82	8.72	6.77	5.97
*Spicara maena*	-	-	-	-	-	-	6.7
*Spicara smaris*	-	-	-	-	10.31	-	-
*Spicara sp*.	-	-	-	-	6.49	-	10.78
*Symphodus tinca*	2.55	-	5.74	-	-	-	-
*Thalassoma pavo*	-	-	4.3	6.29	14.33	4.44	5.78

At Peloponnese, the species that contributed the most to the overall regional ichthyofauna diversity were *Chromis chromis* (Linnaeus, 1758) (31.63%) S. *luridus* 12.98% and *S*. *rivulatus* 11.83%, in total 56.45%. The contribution of most abundant species (11) can be seen at Table 6. At Kefalonia, the most abundant species were S. *luridus* 18.89%, *S*. *rivulatus* 18.89%, and *D*. *vulgaris* 14.2%, in total 51.99% (Table 6). At Lesvos, the most abundant species were *C*. *chromis* (45.72%) and *Symphodus tinca* (Linnaeus, 1758) 11.75%, 57.47% in total. The contribution of most abundant species (6) can be seen at Table 6. At Kalymnos 14 species, see Table 6, are influencing the most the fish fauna diversity in the area, of which *C*. *chromis* (17.43%), *S*. *rivulatus* (10.63%), *S*. *cretense* (Linnaeus, 1758) (8.7%), S. *luridus* (6.43%) and *Thalassoma pavo* (Linnaeus, 1758) (6.35%) are the most significant. At Nisyros, 16 species are contributing the most to the local fish diversity, of which five are most frequent; these are *C*. *chromis* (21.32%), S. *luridus* (8.06%), *S*. *rivulatus* (8.06%), *T*. *pavo* (6.98%) and *S*. *smaris* (Linnaeus, 1758) (6.74%), a total of 57.64% (Table 6). At Samos, only five species were the most abundant, *C*. *chromis* (18.46%), *Sarpa salpa* (Linnaeus, 1758) (11.6%), *S*.*cretense* (8.47%) *Coris julis* (Linnaeus, 1758) (7.33%) and *S*. *rivulatus* (6.56%), in total 52.42%. Table 6 presents the total contribution of most abundant species (14) for Samos sites. Finally, at Santorini the most frequent species were *C*. *chromis* (22.06%), S. *luridus* (10.21%), *S*. *rivulatus* (10.21%), *D*. *sargus* (6.97%) and *S*. *maena* (5.27%), totally 54.72%. At Santorini 16 species (Table 6) were the most influential for the diversity patterns.

**Table 6:** The contribution (%) of the most dominant species among sampling sites, based on the findings of the present study. Marked with yellow are the most dominant species.

Species	Contribution (%)						
	PEL	KEF	LES	KAL	NIS	SAM	SAN
*Anthias anthias*	-	-	-	4.4	-	-	-
*Apogon imberbis*	-	-	-	4.04	-	5.19	2.76
*Boops boops*	-	-	-	-	2.9	3.28	3.73
*Centrolabrus melanocercus*	-	-	-	-	-	2.68	-
*Chromis chromis*	**31.63**	-	**45.72**	**17.43**	**21.32**	**18.46**	**22.06**
*Coris julis*	5.51	-	9.81	5.82	4.41	7.33	4.79
*Diplodus sargus*	4.44	12.46	7.92	6.28	-	6.14	6.97
*Diplodus vulgaris*	5.3	14.2	7.92	5.65	3.78	3.28	4.41
*Epinephelus costae*	-	-	-	-	2.42	-	-
*Mullus surmuletus*	3.22	6.82	-	-	2.67	4.83	2.04
*Oblada melanura*	-	-	-	-	6.5	-	3.54
*Parupeneus forsskali*	-	-	-	4.23	3.42	-	-
*Pterois miles*	-	-	-	3.15	2.42	-	-
*Sarpa salpa*	-	-	-	5.67	-	11.6	-
*Serranus cabrilla*	3.27	-	-	2.11	-	-	2.04
*Serranus scriba*	5.52	11.14	-	-	2.42	3.54	2.04
*Siganus rivulatus*	11.83	**18.89**	-	10.63	8.06	6.56	10.21
*Siganus luridus*	12.98	**18.89**	-	6.43	8.06	4.44	10.21
*Sparisoma cretense*	3.98	13.65	-	8.7	5.4	8.47	4.17
*Spicara maena*	-	-	-	-	-	-	5.27
*Spicara smaris*	-	-	-	-	6.74	-	-
*Spicara sp*.	-	-	-	-	4.77	-	3.54
*Symphodus tinca*	4.63	-	11.75	-	-	-	-
*Thalassoma pavo*	-	-	7.37	6.35	6.98	5.19	4.17

**Fig 2:**
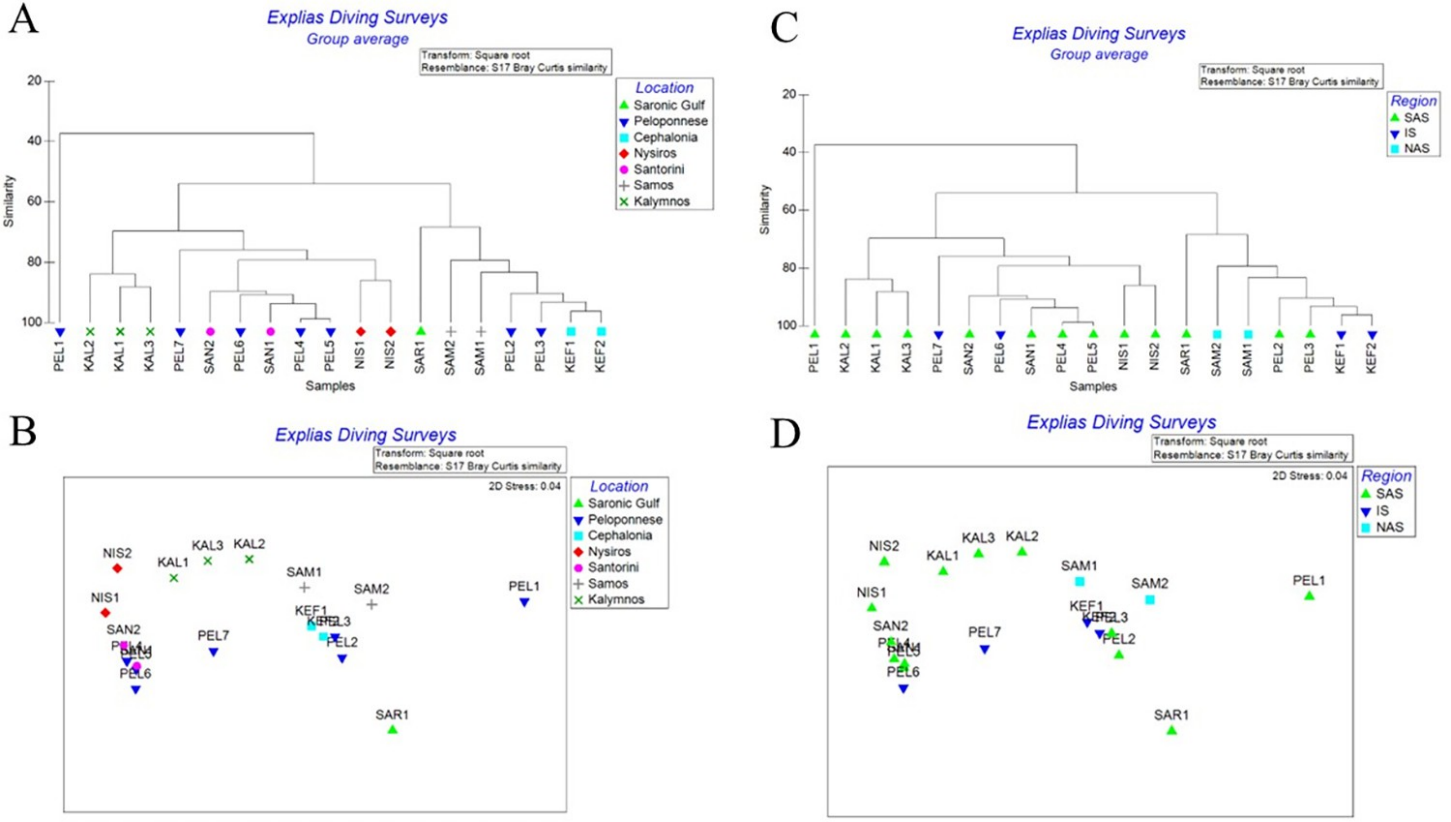
Cluster and MDS analysis of non-indigenous fish fauna. (Α) Cluster analysis (% similarity) of sites and sampling stations (Β) MDS Analysis of sites and sampling stations. (C) Cluster analysis (% similarity) of sites and sampling stations, per ecoregion. (D) MDS Analysis of sites and sampling stations, per ecoregion. (NAS: North Aegean Sea, SAS: South Aegean Sea, IS: Ioanian Sea).

When assessing the differences of species composition and abundances only in respect to the non-native ichthyofauna, the sites and stations grouped differently. The sites PEL4 and PEL5 (Stoupa and Kardamili) are practically identical (95%), and the forming a group with the stations of Santorini Island and the Ionian coasts of Peloponnese, and Nisyros Island (85%). Another distinct group is formed by the diving stations of Kefalonia Island, the Peloponnesian sites PEL2 and PEL3 (Tolo and Neapoli) and the sites of Samos Island >75% and to a lesser extent with Saronikos Gulf (>60%). The stations of Kalymnos Island formed a separate group (>80%). The first station of Peloponnese (PEL1- Kiveri Bay) is the diving site with the least similarity when compared to either the stations or the sites (<40%) (Fig 2).

## Discussion

Although Scientific Diving and Underwater Visual Census are considered to be accurate methodologies to assess fish communities and potential changes in species’ distributions, it may underestimate the presence of *L*. *scelaratus*, as none were recorded in the current study. It seems that the species is highly impacted by the diver-effect (the divers’ presence at the sampling sites), at least during the daytime hours. The diver-effect is a well-known issue that affects different taxa in different ways [60]. As the bibliography suggest, the species dwells in all stations and survey locations. Some biases concerning the population of *F*. *comersonii* were also inducted at the present study. When asking the local people (e.g., diving instructors) on potential diving locations all of them reported that a couple of days earlier or after our survey they encounter some individuals. Moreover, during the surveys in Peloponnese there was a severe and long heatwave (temperatures >45 degrees Celsius), which resulted in the excessive heating of the surface waters. Consequently, at that time many organisms could had migrated temporarily to deeper and colder waters, in which SCUBA diving could not be performed.

Nine non-indigenous species were reported in all regions and sampling sites, excluding Lesvos Island. An important finding of the present study was the high abundances of the genus *Siganus* Forsskål, 1775. The above phenomenon, was previously described off Sicily where dense *S*.*luridus* groups of more than 100 individuals each were observed several times [61, 62]. Both *S*. *luridus* and *S*. *rivulatus* are forming shoals or small groups either mixed with each other or mixed with native species like *S*. *salpa* (Linnaeus, 1758) or/and *S*. *cretense*. It is believed that mixed shoals/groups can be beneficial for the rapid increase in body weight of juveniles when compared to the formation of unmixed groups [63]. The presence and distribution of exotic species at Lesvos Island is underestimated. It is known that both *L*. *sceleratus* and *P*. *miles* occur in the region [64]. Furthermore, it is suggested that the Kalloni Gulf, Lesvos Island, acts as a nursery ground for some alien species like *Penaeus aztecus* Ives, 1891, *Upeneus moluccensis* (Bleeker, 1855) and *Etrumeus golanii* DiBattista, Randall & Bowen, 2012 [64].

An important area for the distribution expansion of non-indigenous species is East Aegean Sea and the south to north axis. Samos Island, though that geographically is included as a region of northern Aegean Sea, based on the present findings it can be said that it “behaves” as a region of the southern Aegean. Both the relative abundances and the body sizes of the recorded lionfishes are very similar to other regions such as the region of Dodecanese, Santorini Island, and some diving sites of Peloponnese. Generally speaking, Peloponnese should be considered as an important region for the expansion of non-indigenous species from Aegean to Ionian and the central Mediterranean basin.

A total of 67 taxa of fish fauna were recorded during the surveys. The current findings present similarities with previous research where both Labridae and Sparidae were the most abundant fish families [61]. We managed to record species with a wide distribution and frequency of occurrence in Greece and the Mediterranean Sea. During the present study, several species with cryptic behaviour and generally shy were recorded. Those species due to their behaviour/ethology are not frequently and systematically recorded especially with the methodology applied in this particular study. Also, we were able to record threatened fish species, included in the International Union for Conservation of Nature’s Red List (https://www.iucnredlist.org/) such as *Epinephelus marginatus* (Lowe, 1834), *Dentex dentex* (Linnaeus, 1758) and species of genus *Thunnus* South, 1845. The most abundant species were *C*. *chromis* and genus Spicara Rafinesque, 1810. That can be explained by the shoaling behaviour of the above taxa [65, 66]. It seems that the formation of shoals besides the widely known advantages of predation avoidance, feeding and reproduction success, it offers other advantages mainly of energy consumption by reducing the metabolic rates [67]. The damselfish, *C*. *chromis*, is among the most valuable fish species of the Mediterranean Sea, providing many services such as nutrient and carbon transfer from the pelagic zone to the littoral, or by representing a key prey item and being a predator for many species [65]. It seems that the invasive lionfish, strongly affects the *C*. *chromis* populations [68] and potentially could threat the species in the future. It is worth to mention that at Nisyros Island we recorded high abundances of *T*. *pavo*. However, most of them regarded fish fry of the species. It is known that *T*. *pavo* shows intense fluctuations at its distribution and those fluctuations are strongly related to the geographic position and to the state of reproduction period [69]. The species (*T*. *pavo*) is an indicator of climate change and the “tropicalization” of the Mediterranean Sea [70]. An interesting finding of the current study is that most of threatened species (groupers, dentex etc.) were amateur individuals, and it seems that the populations of the latter species could face important pressures.

## Conclusions

A brief review of various sources of "grey" literature such as social networks (eg, Facebook©, YouTube©) showed that SCUBA diving during the night hours could be an alternative methodology, because several amateur divers recorded *L*. *sceleratus* individuals. In the present study we avoided performing surveys during the night hours because it was not certain that it would be feasible to do it so in all sampling areas and stations. This would affect the homogeneity of the sampling and results, and therefore of reliable assessments and conclusions. Furthermore, combining different Underwater Visual Census methodologies like the use of transects and lines could result in better estimates of fish biomass and taxa richness, especially for large predatory fish species [71]. Unfortunately, due to the COVID-19 pandemic we were forced to conduct our surveys only during 2021, limiting our dives per site. Our top priority was to include all of our initial diving sites and cover as much space as possible, in respect to the project’s goals.

To obtain more comprehensive results in future research of similar nature, we propose to combine sampling methodologies. For example, they could include scientific diving in the shallower areas, the use of ROV’s (Remote Operated Vehicles), in the same areas and covering deeper regions, and/or the use of bait stations/traps and videography. The above methodologies and techniques are proven to work harmoniously and complement each other and have been used successfully and extensively on numerous occasions [72, 73]. Supplementary to the above, recent studies from the Italian and Greek waters demonstrated that the active involvement of environmentally aware citizens could result in high resolution data either on coastal fish communities [74] or on the detailed distribution and habitat preferences of threatened taxa [75]. In addition, obtaining samples either for morphological or/and for molecular studies would be more than useful and complementary to any field-based study like the current.

## Supporting information

S1 File (XLSX)
